# Correlation of negative emotion, fatigue level and internet addiction in college students: implication for coping strategies

**DOI:** 10.1186/s12888-024-05711-5

**Published:** 2024-04-09

**Authors:** Shanshan Gu, Xue Min, Jing Xu, Shu Chen

**Affiliations:** 1 Zhejiang Business College, 310053 Hangzhou, Zhejiang China; 2 Center for Rehabilitation Medicine, Department of Rehabilitation, Comprehensive Rehabilitation Ward, Affiliated People’s Hospital, Zhejiang Provincial People’s Hospital, Hangzhou Medical College, No. 158 Shangtang road, Gongshu district, 310024 Hangzhou, Zhejiang Province China

**Keywords:** Emotion, Fatigue, Internet addiction, College, Student, Care, Management

## Abstract

**Background:**

Internet addiction has an important influence on the development of physical and mental health of college students. The purpose of this study is to evaluate the current status and the correlation between college students’ negative emotion, fatigue level and Internet addiction disorder, and to provide reference for the care and management of college students.

**Methods:**

We conducted a questionnaire survey on a cluster sample of college students from October to November 15, 2022. Internet addiction scale, fatigue assessment scale and positive and negative emotion scale were used for survey. Pearson correlation analysis and mediating effect test were performed to analyze the correlation and effects.

**Results:**

A total of 1546 valid questionnaires were collected. The incidence of internet addiction in college student was 20.38%. The total score of internet addiction was 52.94 ± 12.47, the total fatigue score was 69.27 ± 3.19, the score of positive emotion of college students was 31. 41 ± 5.09, and the negative emotion score was 18.54 ± 5.68. The total score of internet addiction were positively correlated with score of negative emotion (all *P* < 0. 05). The total score of internet addiction scale of college students were positively correlated and each factor score of with the score of fatigue severity (all *P* < 0. 05). Fatigue played an intermediary role in the prediction of negative emotion and internet addiction of college students, with an intermediary role of-0.433, accounting for 76.35% of the total effect.

**Conclusion:**

The college students’ positive emotion may be strengthened to reduce their fatigue level and negative emotion so as to reduce internet addiction.

## Background

With the rapid popularization and development of the internet, people begin to pay attention to the social reality problem of internet addiction, especially adolescent internet addiction [1]. College students or teenagers are susceptible to internet addiction. The survey results [2] show that 20.6% of college students are at risk of internet addiction, the surveyed college students use mobile phones for an average of 7 to 9 h a day, with an average of 118 mobile phones per person per day. Previous researches [3, 4] show that the proportion of college students with internet addiction is as high as 16. 5%. 6% ∼ 29.5%. Previous studies [5, 6] have found that internet addiction can damage people’s executive control ability, emotional recognition ability and central integration ability. Internet addicts are more likely to process negative social cognition [7]. Teenagers with negative withdrawal tendencies are also more likely to have internet addiction [8]. Therefore, the prevention and care of internet addiction are vital to the health development of teenagers.

Under the sustained consumption of cognitive and emotional will, people may experience a decline in psychological function and subjective experience of fatigue and tiredness [9, 10]. Therefore, emotion, especially negative emotion, is easy to make teenagers escape into the world of the internet [11]. Previous studies [12, 13] have reported that long-term excessive use of the internet has an impact on the mental health of adolescents, the emotions and fatigue may be closely associated with internet addiction. Currently, there are very few studies on the correlation of negative emotion, fatigue level and internet addiction in college students. Therefore, we aimed to conduct a cross-sectional study to evaluate the current status and potential correlation of negative emotion, fatigue level and internet addiction in college students. This study assumed that fatigue played an intermediary role between college students’ emotions, especially negative emotions and internet addiction.

## Methods

This cross-sectional survey was approved by the Ethics Committee of the University (Ethics approval number: 2022zsy-kj-11). Written informed consents had been obtained from all the included students. According to previous report [14], the routine sample size was determined by 10 ∼ 20 times of the analytical factor and this study required more than 56 items × 20 = 1120 samples. In order to reduce the error and expand by 30% on this basis, this study needed at least about 1456 students.

From October to November 15, 2022, we conducted a questionnaire survey on a cluster sample of college students in a university in Hangzhou, Zhejiang province, China. We randomly selected the corresponding number of students according to the proportion of college students in each grade. The inclusion criteria of the students were as follows: full-time college students; they were currently studying normally in our college; they volunteered to participate in this study.

All the questionnaires in this study were investigated anonymously. The questionnaires used are as follows: (1) We designed a general information questionnaire for medical students, including medical students’ age, gender, body mass index (BMI), whether the student was the only child of family, parents’ educational level. (2) Internet addiction scale [15]: In this scale, 26 questions were assessed with 4 grades, including tolerance symptom, withdrawal symptom, forced to surf the Internet, interpersonal health, time management. The higher the score, the more serious respondents are addicted to the Internet. The scale was widely used in the study of Internet addiction with good reliability and validity [16]. If the total score of the internet addiction scale was ≥ 63, the respondents are assessed to have internet addiction. The construct validity of the scale was good (comparative fit index (CFI) = 0. 96), and the internal consistency coefficient of the scale was 0.91 [17]. (3) Fatigue assessment scale (FAI) [18]: There are 29 declarative sentences, which are scored from 1 to 7 and scored at seven points, including four subscales: the severity of fatigue, the environmental specificity of fatigue, the results of fatigue and the response of fatigue to rest and sleep. the higher the score, the higher the degree of fatigue. It has been reported that FAI is easy to operate and can accurately evaluate the degree and characteristics of fatigue. The construct validity of the scale was good (CFI = 0. 95), and the internal consistency coefficient of FAI scale was 0.93 [19]. (4) Positive and negative emotion scale (PANAS) [20]: This scale was compiled by Watson and Clark of South Meaddist University in 1988 to evaluate individuals’ positive and negative emotions. The Chinese version of the positive and negative emotion scale was translated and verified by Huang Li et al. The scale contains 20 adjectives reflecting emotion, and 10 words correspond to positive and negative emotion factors respectively. The two factors were statistically scored, in which the higher the score of positive emotion, the more positive emotion and more concentration. On the contrary, the higher the score of negative emotion is, the more painful it is, and the more negative emotion is. The construct validity of the PANAS scale was good (CFI = 0. 88), and the internal consistency coefficient of the scale was 0.97 [21].

### Survey procedures

Before collecting the questionnaire, we adjusted the instruction of the questionnaire and the answer format of the questionnaire to minimize the resistance and fatigue of the students when filling out the questionnaire, and to ensure that the subjects answered the questions carefully on the basis of understanding the meaning of the questions. We introduced the purpose of this study to students, and emphasized that this questionnaire answered anonymously, abided by the principle of confidentiality, the data collected was only for scientific research, there was no difference between right and wrong, and they can choose according to their own real situation. The filling time of the questionnaire was limited to 20 min, and the surveyors checked the questionnaire data on the spot. If there were missing data, the students were required to fill in the questionnaire. If the students were unwilling, the questionnaire would be invalidated.

### Statistical analysis

In this study, SPSS 22.0 was used to analyze the data, the counting data were expressed by case and frequency, and the measurement data were expressed by mean ± standard deviation. Independent sample t-test was used to compare the measurement data between the two groups. To understand the potential correlation and interaction of negative emotion, fatigue level and internet addiction in college students, Pearson correlation analysis was used to analyze the relationship between groups of measurement data to ensure the feasibility of subsequent testing of hypothetical model fitting. And mediating effect test and Bootstrap method were used to analyze the intermediary role of fatigue in negative emotion and internet addiction. In this study, *P* < 0.05 indicating that the difference between groups was statistically significant.

## Results

Initially 1580 questionnaires were distributed and a total of 1546 valid questionnaires finally were collected. The characteristics of included college students are presented in Table 1.

**Table 1:** The characteristics of included college students(*n* = 1546)

Characteristics	Cases	Percentage (%)
Gender		
Female	989	63.97%
Male	557	36.03%
Age(y)		
< 20	361	23.35%
20 ∼ 25	1044	67.53%
> 25	141	9.12%
BMI(kg/m^2^)		
< 20	257	16.62%
20 ∼ 25	1094	70.76%
>25	195	12.61%
Only child of family		
Yes	982	63.52%
No	564	36.48%
Parents’ educational level		
Primary school	107	6.92%
Junior high school	405	26.20%
Senior high school	881	56.99%
University	153	9.89%

BMI, body mass index

As shown in Table 2, the total score of internet addiction was 52.94 ± 12.47, 315 students’ internet addiction score ≥ 63, the incidence of internet addiction in college student was 20.38%.

**Table 2:** The internet addiction scores of included college students

Items	Average scores
Tolerance symptom	8.46 ± 2.44
Withdrawal symptom	9.92 ± 2.81
Forced to surf the Internet	9.66 ± 2.96
Interpersonal health	13.79 ± 4.34
Time management	9.35 ± 2.91
Total score of internet addiction	52.94 ± 12.47

As shown in Table 3, the total fatigue score was 69.27 ± 3.19, it showed that the fatigue level of college students was in the middle level.

**Table 3:** The fatigue scores of college students

Items	Average scores
Severity of fatigue	32.68 ± 12.32
Environmental specificity of fatigue	25.91 ± 7.09
Results of fatigue	12.75 ± 4.22
Response of fatigue to rest sleep	11.88 ± 2.43
The total fatigue score	69.27 ± 3.19

The score of positive emotion of college students was 31. 41 ± 5.09, and the negative emotion score was 18.54 ± 5.68. As shown in Table 4, the total score and each factor score of internet addiction score of college students were negatively correlated with the score of positive emotion (all *P* < 0. 05), the total score of internet addiction scale and the scores of all factors were positively correlated with the score of negative emotion (all *P* < 0. 05). The total score and each factor score of internet addiction scale of college students were positively correlated with the score of fatigue severity (all *P* < 0. 05).

**Table 4:** Correlation analysis of college students’ internet addiction scale score, fatigue score and emotional score

Items	Positive emotional score	Negative emotional score	Severity of fatigue	Environmental specificity of fatigue	Results of fatigue	Response of fatigue to rest sleep	The total fatigue score
Total score of internet addiction	-0.346^*^	0.422^*^	0.038^*^	0.021	0.029^*^	0.005	0.036^*^
Tolerance symptom	-0.289^*^	0.357^*^	0.034^*^	0.027^*^	0.038^*^	0.005	0.042^*^
Withdrawal symptom	-0.265^*^	0.371^*^	0.032^*^	0.017	0.025^*^	0.006	0.045^*^
Forced to surf the Internet	-0.322^*^	0.398^*^	0.045^*^	0.020	0.029^*^	0.004	0.038^*^
Interpersonal health	-0.314^*^	0.385^*^	0.029^*^	0.014	0.019	0.003	0.029^*^
Time management	-0.296^*^	0.336^*^	0.032^*^	0.037^*^	0.028^*^	0.026^*^	0.044^*^

^*^, *P* < 0.05

**Fig. 1:**
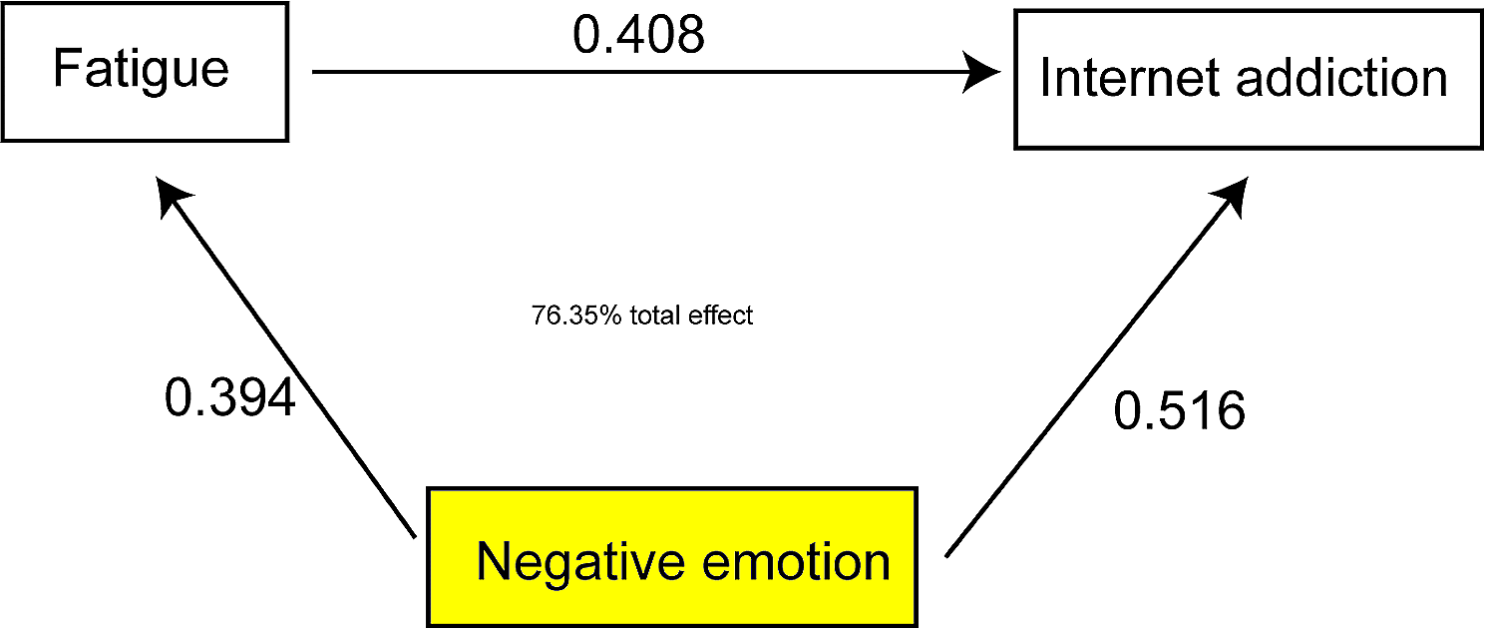
The chart on the mediating effect of fatigue on negative emotion and internet addiction of college students

As shown in Fig. 1; Table 5, fatigue played an intermediary role in the prediction of l negative emotion and internet addiction of college students, with an intermediary role of-0.433, accounting for 76.35% of the total effect.

**Table 5:** The mediating effect of fatigue on the scores of negative emotion and internet addiction of college students

Influencing path	Standardized effect size	Standard error	Percentage of the total effect	95%CI	
				Upper limit	Lower limit
Total effect	-0.603	0.054	-	-0.667	-5.321
Direct effect	-0.284	0.032	23.65%	-0.309	-0.196
Mediating effect	-0.433	0.058	76.35%	-0.502	-0.395

## Discussions

With its unique advantages and speed of development, the network is changing our way of working, learning and thinking, permeating every corner of our daily life, and bringing us into a new era. However, the network is a “double-edged sword”. Its negative effect is the same as its positive effect, which involves all aspects of social life [22, 23]. For contemporary college students, the Internet has become an important means of learning knowledge, finding information, chatting, making friends, satisfying personal hobbies and understanding current events [24]. How to give full play to the positive role of the Internet, avoid its negative effects as far as possible, minimize the Internet addiction behavior of college students, and put forward intervention measures according to its influencing factors and possible consequences, it is the focus of this study to provide a basis for medical departments, education departments, parents, college students and relevant departments of society to build network civilization [25]. The results of this study show that college students with more positive emotions are not prone to internet addiction, while college students with more negative emotions are prone to internet addiction. The more serious the fatigue is, the more likely it is to become internet addictive. And fatigue as an intermediary factor will further deepen the impact of negative emotions on internet addiction.

Fatigue is the response of the body to long-term continuous activity or mental load, it can affect the nervous and endocrine system, increases the secretion of epinephrine and cortisol hormones, which have a direct negative effect on mood [26]. In addition, fatigue also reduces the body’s absorption of folic acid and vitamin B12, which play an important role in the body’s metabolism and cell growth [27, 28]. In addition, fatigue can cause different degrees of physiological reactions, such as accelerated heartbeat, elevated blood pressure, shortness of breath, etc., making people feel irritable, anxious, or depressed, which in turn exacerbates the feeling of fatigue [29]. Another condition that is easy to detect is that fatigue can affect the quality of sleep, which in turn aggravate the sense of fatigue, fall into a bad cycle that ca. not break free, and even lead to mental illness in the long run [30, 31]. Therefore, the early intervention of fatigue is of great significance.

It has found that when the individual is in a state of mental fatigue, the negative emotion usually increases and it is difficult to concentrate [32]. When facing negative emotions, college students need to carry out emotional regulation, and emotional regulation will damage college students’ limited resources of self-control, which is a kind of ability to control and restrain their own emotions. People’s self-control, like muscles, has an upper limit, when beyond this limit, people’s behavior is easy to get out of control [33–35]. According to the theory of limited self-control, the loss of self-control will lead to the decline of college students’ ability to resist hedonistic experiences, so they are prone to internet addiction [36, 37].

The basic explanation of the loss of compensation hypothesis for internet addiction is that internet rational compensation leads to loss of compensation and Internet addiction behavior [38]. Both negative emotion and fatigue will increase the loss of self-control of college students, and college students will follow the hypothesis of loss of compensation to try to get entertainment and relaxation from the Internet, but in fact, the loss of self-control makes college students unable to better control their online behavior, which is easy to lead to internet addiction, and excessive use of the internet makes the brain highly tense for a long time, which is easy to make people tired [39–41]. Fatigue further stimulates internet addiction, which forms a vicious circle. This study supports the views of previous studies [42–44] on the relationship between emotion and Internet addiction. Therefore, in order to reduce the formation of college students’ internet addiction and reduce the impact of negative emotions on college students’ internet addiction, solving college students’ fatigue may be the key question, and college students need to learn to care for themselves and be kind to themselves. And previous studies [45–47] have also found that the incidence of suicidal ideation in patients with internet addiction will increase, so the phenomenon of internet addiction among college students needs to attract further attention from college students themselves, their families, schools and society.

Prevention first, early intervention should be a coping strategy to reduce college students’ internet addiction. As an important work, we should focus on finding high-risk groups to provide more assistance. It includes goal setting, self-suggestion and reminder, aversion therapy, diversion, making a personal goal list, and so on. Students with addictive tendencies are grouped into long and small groups to adjust and improve their relations with others, learn new attitudes and behaviors, reduce fatigue levels, and correctly understand and treat life in the form of group counseling [48, 49]. Besides, it is necessary to establish a linkage between schools and professional institutions, correctly identify patients with serious internet addiction, and refer them to professional medical institutions for active treatment [50, 51]. At present, the comprehensive treatment of drug therapy combined with psychological counseling has been widely used in clinic, and the practice has proved to be an effective treatment measure [52].

This study reveals the mechanism of fatigue and negative emotion on college students’ internet addiction, but there are some limitations need further consideration. First of all, the sample of this study comes from a single university, the sample size is small, and the sample needs to be expanded to evaluate the generalizability of the findings. Secondly, this study is a questionnaire survey, which cannot prove the causal relationship, the findings should be treated with cautions. Follow-up researches using the experimental design method or longitudinal studies are needed to understand the mechanism of college students’ internet addiction disorder and elucidate causality in the future.

## Conclusions

In summary, this study has found that the phenomenon of internet addiction among college students is more serious. Negative emotion not only directly affects the degree of internet addiction of college students, but also can affect the degree of internet addiction of college students through the intermediary effect of fatigue. Colleges and universities may strengthen network education and management, adopt various forms to widely carry out network knowledge publicity, network psychology lectures, network psychology square consultation and other activities, so that students and counselors can identify internet addiction and understand intervention measures. Psychological counselors with addictive tendencies should carry out systematic and long-term psychological intervention, adopt the combination of collective psychological counseling and individual psychological counseling, and pass through professional counselors to help visitors know themselves, accept themselves, appreciate themselves, overcome growth obstacles and develop their personal potential. School administrators and teachers should strengthen the guidance and construction of college students’ positive emotion and reduce their fatigue level and negative emotion so as to reduce the occurrence of internet addiction.

## Data Availability

All data generated or analyzed during this study are included in this published article.

## Abbreviations

BMI: body mass index
FAI: fatigue assessment scale
PANAS: positive and negative emotion scale

## References

[1] Dawadi P, Khadka S, Maharjan S, Baniya A, Khadka S, Thapa S, Deo R (2022). Internet addiction among Undergraduate Medical students of a Medical College: a descriptive cross-sectional study. JNMA J Nepal Med Assoc.

[2] Gao S, Zeng F, Chen F (2022). Current situation and influencing factors of internet addiction among College students in higher Vocational Colleges– A case study of colleges and universities in Hunan Province. J Huaihua Univ.

[3] Zhang W, Xu R. Effect of Exercise Intervention on Internet Addiction and Autonomic Nervous Function in College Students. *Biomed Res Int* 2022, 2022:5935353. 10.1155/2022/5935353 PMC9467718 36105927

[4] Gavurova B, Ivankova V, Rigelsky M, Mudarri T (2022). Internet addiction in Socio-Demographic, Academic, and Psychological Profile of College Students during the COVID-19 pandemic in the Czech Republic and Slovakia. Front Public Health.

[5] Arslan G, Coskun M (2022). Social Exclusion, Self-Forgiveness, Mindfulness, and internet addiction in College students: a Moderated Mediation Approach. Int J Ment Health Addict.

[6] Kumar G, Dash P, Jnaneswar A, Suresan V, Jha K, Ghosal S (2022). Impact of internet addiction during COVID-19 on anxiety and sleep quality among college students of Bhubaneswar city. J Educ Health Promot.

[7] Xie Y, Wu J, Zhang C, Zhu L (2022). Cumulative childhood trauma and cybervictimization among Chinese college students: internet addiction as a mediator and roommate relationships as a moderator. Front Psychol.

[8] Yang W, Hu W, Morita N, Ogai Y, Saito T, Wei Y. Impact of short-term intensive-type cognitive behavioral therapy intervention on internet addiction among Chinese College students: a Randomized Controlled Trial. Int J Environ Res Public Health 2022, 19(9). 10.3390/ijerph19095212 PMC9099546 35564611

[9] Pan L, Li J, Hu Z, Wu H. The Effect of COVID-19 perceived risk on internet addiction among College students in China: an empirical study based on the Structural equation Model. Int J Environ Res Public Health 2022, 19(20). 10.3390/ijerph192013377 PMC9603053 36293960

[10] Jiang M, Zhao Y, Wang J, Hua L, Chen Y, Yao Y, Jin Y (2022). Serial multiple mediation of the correlation between internet addiction and depression by Social Support and Sleep Quality of College Students during the COVID-19 epidemic. Psychiatry Investig.

[11] Du Z, Zhang X (2022). Analysis of the mediating effects of self-efficacy and self-control between physical activity and internet addiction among Chinese college students. Front Psychol.

[12] Miao F (2022). Analysis of college students’ social network addiction and school mental health education. Educational Res.

[13] Jin N, Lu W, Song M (2023). Analysis of current situation and influencing factors of internet addiction among medical students. Neurol Dis Mental Health.

[14] Weijun Z, Fan H (2020). The method of calculating the sample size of current situation survey. Prev Med.

[15] Ko CH, Yen JY, Chen SH, Yang MJ, Lin HC, Yen CF (2009). Proposed diagnostic criteria and the screening and diagnosing tool of internet addiction in college students. Compr Psychiatry.

[16] Jianqin C, Jinwei Y, Jun Y (2010). Reliability and validity of internet addiction impairment scale. Chin Gen Med.

[17] Sulan L, Wangqiao Z, Xiaodong L (2021). Internet addiction of medical students in traditional Chinese medicine colleges and universities in Jiangxi Province and its influencing factors. Chin J Health Psychol.

[18] Zhang Z. Handbook of behavioral medical scales. Volume 16. Beijin; 2005.

[19] Shengli L, Hongzhan Q, Wenjun Y (2010). Investigation and analysis of nurses’ fatigue and perceived social support. Chin J Practical Nurs.

[20] Watson D, Clark LA, Tellegen A (1988). Development and validation of brief measures of positive and negative affect: the PANAS scales. J Pers Soc Psychol.

[21] Lin Q, Xue Z, Yanfei W (2008). Revision of positive emotion and negative emotion scale (PANAS). Appl Psychol.

[22] Chang B, Hou J (2022). The Association between Perceived Risk of COVID-19, psychological distress, and internet addiction in College students: an application of stress process model. Front Psychol.

[23] Zhao Y, Zhang K, Griffiths MD (2022). Serial mediation roles of Alexithymia and Loneliness in the association between family function and internet addiction among Chinese College Students. Front Psychol.

[24] Liang S, Ren Z, Yang G (2022). Cross-sectional and prospective association between internet addiction and risk of fatigue among Chinese college students. Med (Baltim).

[25] Ibrahim AK, Fouad I, Kelly SJ, El Fawal B, Ahmed GK (2022). Prevalence and determinants of internet addiction among medical students and its association with depression. J Affect Disord.

[26] Rzepka M, Chmiela T, Kaczmarczyk A, Krzystanek E. Insomnia, fatigue, bladder disorders and Mood disorders among Polish patients with multiple Sclerosis: cross-sectional study. J Clin Med 2024, 13(4). 10.3390/jcm13041043 PMC10888699 38398356

[27] Tornero-Aguilera JF, Jimenez-Morcillo J, Rubio-Zarapuz A, Clemente-Suarez VJ. Central and peripheral fatigue in physical Exercise explained: a narrative review. Int J Environ Res Public Health 2022, 19(7). 10.3390/ijerph19073909 PMC8997532 35409591

[28] Davis MP, Walsh D (2010). Mechanisms of fatigue. J Support Oncol.

[29] Masel BE, Zgaljardic DJ, Forman J (2017). Post-traumatic hypopituitarism and fatigue. Neuropsychol Rehabil.

[30] Tackey C, Slepian PM, Clarke H, Mittal N (2023). Post-viral Pain, fatigue, and Sleep Disturbance syndromes: current knowledge and future directions. Can J Pain.

[31] Booker LA, Fitzgerald J, Mills J, Bish M, Spong J, Deacon-Crouch M, Skinner TC (2024). Sleep and fatigue management strategies: how nurses, midwives and paramedics cope with their shift work schedules-a qualitative study. Nurs Open.

[32] Lin SC, Tsai KW, Chen MW, Koo M (2013). Association between fatigue and internet addiction in female hospital nurses. J Adv Nurs.

[33] Buneviciene I, Bunevicius A (2021). Prevalence of internet addiction in healthcare professionals: systematic review and meta-analysis. Int J Soc Psychiatry.

[34] Ohayon MM, Roberts L (2021). Internet gaming disorder and comorbidities among campus-dwelling U.S. university students. Psychiatry Res.

[35] Fu SC, Pang NTP, Wider W. Relationship between internet addiction, personality factors, and emotional distress among adolescents in Malaysia. Child (Basel) 2022, 9(12). 10.3390/children9121883 PMC9776460 36553330

[36] Son HG, Cho HJ, Jeong KH. The Effects of Korean Parents’ Smartphone Addiction on Korean Children’s Smartphone Addiction: Moderating Effects of Children’s Gender and Age. Int J Environ Res Public Health 2021, 18(13). 10.3390/ijerph18136685 PMC8297017 34206185

[37] Hwang IW, Choe JP, Park JH, Lee JM. Association between physical activity, sedentary behavior, satisfaction with sleep fatigue recovery and smartphone dependency among Korean adolescents: an age- and gender-matched study. Int J Environ Res Public Health 2022, 19(23). 10.3390/ijerph192316034 PMC9739357 36498107

[38] Qiong W, Tao X, Huiying L (2019). The relationship between parental rejection and internet addiction of left-behind children: a moderated intermediary model. Psychol Dev Educ.

[39] Qian D, Yongxin Z, Hua W (2019). The doctrine of the mean and college students’ internet addiction: the sequence mediation of social support and loneliness. Psychol Behav Res.

[40] Kumari R, Langer B, Gupta R, Gupta RK, Mir MT, Shafi B, Kour T, Raina SK (2022). Prevalence and determinants of internet addiction among the students of professional colleges in the Jammu region. J Family Med Prim Care.

[41] Fan Z, Chen M, Lin Y (2022). Self-control and problematic internet use in College students: the Chain Mediating Effect of rejection sensitivity and loneliness. Psychol Res Behav Manag.

[42] Karaer Y, Akdemir D (2019). Parenting styles, perceived social support and emotion regulation in adolescents with internet addiction. Compr Psychiatry.

[43] Younes F, Halawi G, Jabbour H, El Osta N, Karam L, Hajj A, Rabbaa Khabbaz L (2016). Internet Addiction and relationships with Insomnia, anxiety, Depression, stress and self-esteem in University students: a cross-sectional designed study. PLoS ONE.

[44] Ahmed GK, Abdalla AA, Mohamed AM, Mohamed LA, Shamaa HA (2022). Relation between internet gaming addiction and comorbid psychiatric disorders and emotion avoidance among adolescents: a cross-sectional study. Psychiatry Res.

[45] Yunjiao Z, Yehuan S, Jiahu H (2019). Relationship between negative life event depression and internet addiction disorder among freshmen in vocational colleges in Anhui Province. School Health China.

[46] Shaolan W, Yongxin Y, Chunmei W. Path analysis of predisposing factors of suicidal ideation in patients with internet addiction. Chin J Behav Med Brain Sci 2018 27(3):216–21.

[47] Sayed M, Naiim CM, Aboelsaad M, Ibrahim MK (2022). Internet addiction and relationships with depression, anxiety, stress and academic performance among Egypt pharmacy students: a cross-sectional designed study. BMC Public Health.

[48] Yang SY, Wang YC, Lee YC, Lin YL, Hsieh PL, Lin PH. Does Smartphone Addiction, Social Media Addiction, and/or Internet Game Addiction Affect Adolescents’ Interpersonal Interactions? Healthc (Basel) 2022, 10(5). 10.3390/healthcare10050963 PMC9141886 35628099

[49] Kamal S, Kamal S, Mubeen SM, Shah AM, Samar SS, Zehra R, Khalid H, Naeem R (2022). Smartphone addiction and its associated behaviors among medical and dental students in Pakistan: a cross-sectional survey. J Educ Health Promot.

[50] He Z, Li M (2022). Executive function and Social Media Addiction in Female College students: the mediating role of affective state and stress. J Genet Psychol.

[51] Deb N, Roy P (2022). Internet addiction, depression, anxiety and stress among first year medical students after COVID-19 lockdown: a cross sectional study in West Bengal, India. J Family Med Prim Care.

[52] Liu H, Zhou Z, Zhu E, Huang L, Zhang M (2022). Smartphone addiction and its associated factors among freshmen medical students in China: a cross-sectional study. BMC Psychiatry.

